# Effects of Fat and Protein Levels on Foraging Preferences of Tannin in Scatter-Hoarding Rodents

**DOI:** 10.1371/journal.pone.0040640

**Published:** 2012-07-10

**Authors:** Bo Wang, Jin Chen

**Affiliations:** 1 Key Laboratory of Tropical Forest Ecology, Xishuangbanna Tropical Botanical Garden, Chinese Academy of Sciences, Mengla, Yunnan, China; 2 Graduate School of Chinese Academy of Sciences, Beijing, China; University of Jyväskylä, Finland

## Abstract

Both as consumers and dispersers of seeds, scatter-hoarding rodents often play an important role in the reproductive ecology of many plant species. However, the seeds of many plant species contain tannins, which are a diverse group of water-soluble phenolic compounds that have a high affinity for proteins. The amount of tannins in seeds is expected to affect rodent foraging preferences because of their major impact on rodent physiology and survival. However, variable results have been obtained in studies that evaluated the effects of tannin on rodent foraging behavior. Hence, in this study, we aimed to explain these inconsistent results and proposed that a combination of seed traits might be important in rodent foraging behavior, because it is difficult to distinguish between the effects of individual traits on rodent foraging behavior and the interactions among them. By using a novel artificial seed system, we manipulated seed tannin and fat/protein levels to examine directly the univariate effects of each component on the seed preferences of free-ranging forest rats (*Apodemus latronum* and *Apodemus chevrieri*) during the behavioral process of scatter hoarding. Our results showed that both tannin and fat/protein had significant effects on rodent foraging behavior. Although only a few interactive effects of tannin and fat/protein were recorded, higher concentrations of both fat and protein could attenuate the exclusion of seeds with higher tannin concentrations by rodents, thus influencing seed fate. Furthermore, aside from the concentrations of tannin, fat, and protein, numerous other traits of plant seeds may also influence rodent foraging behavior. We suggest that by clarifying rodent foraging preferences, a better understanding of the evolution of plant seed traits may be obtained because of their strong potential for selective pressure.

## Introduction

Scatter-hoarding rodents often play an important role in the dispersal of seeds for some plant species because of their high relative abundance and ubiquity [1]. In addition, acting as both consumers and dispersers of seeds, rodents also play an important role on seedling regeneration, colonization ability, spatial distribution, and the reproductive ecology of trees [1], [2]. Understanding seed preference in scatter-hoarding rodents is complicated because selection involves a complex decision-making process, including both spatial and temporal aspects. Hence, the potential future value of seeds may influence current foraging decisions by rodents. Seed preference in rodents therefore encompasses preference for (1) seeds to eat *in situ* versus (2) seeds to scatter hoard, as well as decisions on where and how far seeds should be transported. Consequently, seed traits have been considered as one of the most essential factors that influence foraging preferences by scatter-hoarding rodents, which in turn regulate seed fate [2]–[7].

Tannins are widely distributed in the seeds of various plant species [5], [8]. They are a diverse group of water-soluble phenolic compounds with high affinity for proteins. As a series important plant secondary chemicals, tannins in seeds are believed to influence rodent foraging preference, because of their severe effects on rodent physiology and survival [9]–[11]. Many studies have discussed the effects of tannin on rodent foraging behavior and, in turn, seed fate. However, the results of these studies are inconsistent. For example, some studies have suggested that rodents prefer to cache seeds with high tannin levels and consume seeds with low tannin levels [4], [12], [13], whereas other studies found several different, even opposite results [5], [7], [11]. Wang and Chen (2009) suggested that the foraging behavior of scatter-hoarding rodents in response to tannin content might be a multivariate response to different environment factors where experiments have been conducted [7]. However, recent experiments by these authors have demonstrated that environmental factors, such as seed abundance and tannin content levels of available food in the environment, have no effect on the foraging behavior of rodents on tannin [11]. Here, we propose an alternative theory, whereby overall seed traits influence rodent foraging preference because all seed traits are combined, and it is difficult to distinguish individual trait effects on rodents foraging behavior or the interactions among them.

Fat and protein are 2 key nutrients in mammal diets, and hence comprise inevitable effective factors during rodent foraging processes [5], [14]–[17]. Many studies have discussed the possible effects of fat and/or protein on rodent foraging preferences for seeds with different tannin content levels [5], [12], [17]. However, these studies could not distinguish the relative effects of protein and/or fat, because different species of natural seeds that had different seed traits were used. Hence, the use of artificial seeds provides an opportunity to test the effect of each specific trait on rodent foraging behavior, while maintaining other traits at constant levels [7], [8]. To our knowledge, only two studies [18], [19], have used artificial seeds in an attempt to examine the effects of both tannin and fat/protein on foraging preferences in free-ranging scatter-hoarding rodents, with very interesting results. Smallwood and Peter (1986) found that the addition of tannin significantly reduced the probability of an artificial seed being eaten and the length of time spent eating it, while the addition of fat was shown to attenuate the effects of tannin [18]. Barthelmess (2001) found that grey squirrels consistently preferred seeds with low tannin content; however, they could not distinguish between seeds with low and high protein content levels, and the intensity of preference for foods with low tannin content varied across seasons [19]. However, these 2 studies only presented the harvesting preference of rodents and did not provide any seed fate results, e.g., whether the seeds were eaten *in situ* or cached and how far the cached seeds were dispersed.

To further explore how seed fat and protein concentrations influence scatter-hoarding rodent preferences on seed tannin concentrations during their whole foraging process, we manipulated seed fat, protein, and tannin content levels by using an artificial seed system similar to that developed in our previous study [7], [11]. In this study, we separately evaluated the influence of each specific trait on seed preference during the process of foraging by scatter-hoarding rodents. In addition, we recorded the time (days) to seed harvest, seeds removal vs. being eaten *in situ*, and distance of seeds carried by rodents. We tested the following hypotheses: (1) seeds with low tannin and/or high fat/protein content are more likely to be harvested by rodents than seeds with high tannin and low fat/protein content; (2) seeds with low tannin and/or high fat/protein content are harvested more quickly by rodents; (3) seeds with low tannin and/or high fat/protein content are carried to further distances by rodents; (4) both fat and protein might attenuate the exclusion of seeds with higher tannin concentrations by rodents, thus influencing seed fate.

## Results

### Rodent Survey

In total, 18 individuals (7 *Apodemus latronum* and 11 *Apodemus chevrieri*) and 29 individuals (18 *A. latronum* and 11 *A. chevrieri*) were caught in the traps during the summer and autumn, respectively. Trap success was much higher in autumn than in summer (12.2% vs. 5.8%, *P*<0.05, chi-square test). However, the same 2 rodent species were captured in both seasons, with no significant difference in composition (*P*>0.05, chi-square test).

### General Comparison of Rodent Foraging on Artificial Seeds between Seasons

**Figure 1:**
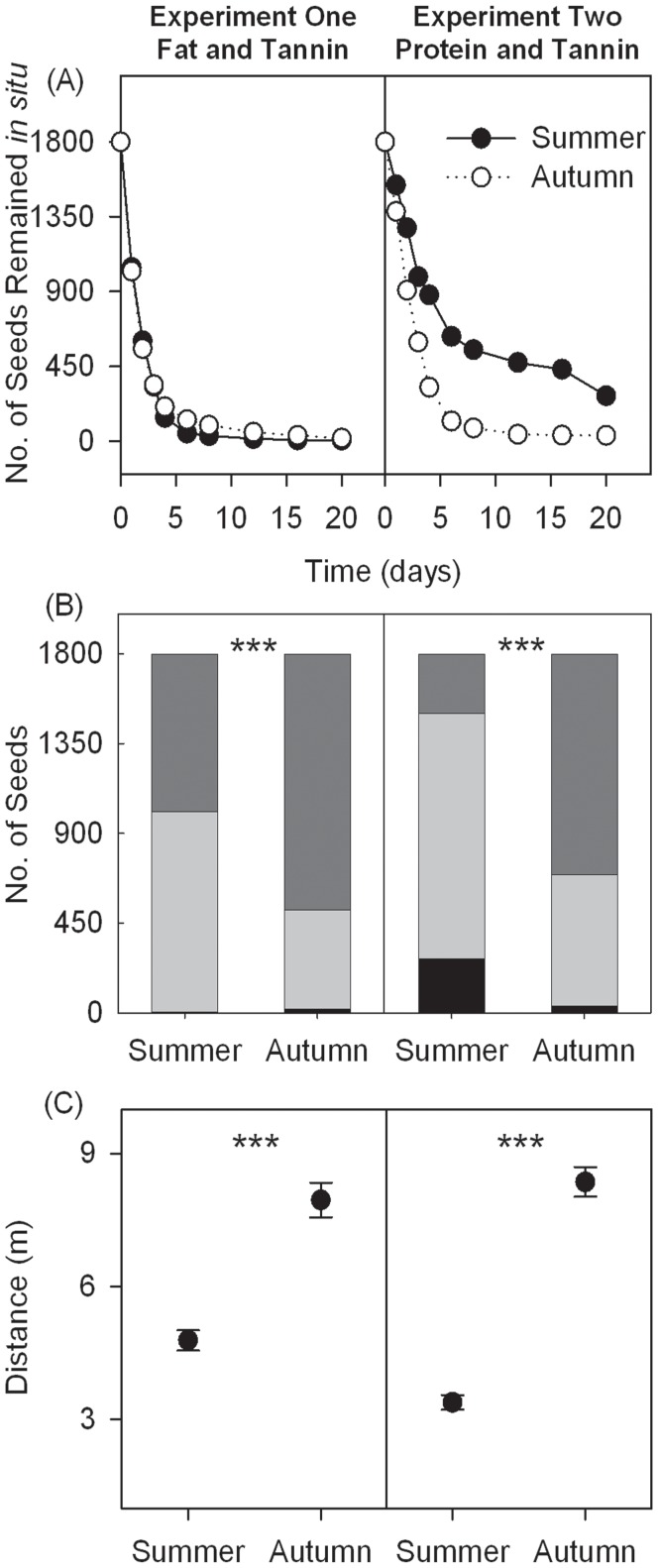
General comparison of rodent foraging preferences for artificial seeds between seasons in both experiments. (A) The time to seed harvest of 1800 seeds after placement at seed releasing plots. (B) Differences in seed fates between seasons. Black bars represent the seeds left intact *in situ*, light grey bars represent seeds eaten *in situ*, while dark gray bars represent seeds removed by rodents. (*** stands for significant difference, chi-squared test, P<0.001). (C) Differences in dispersal distance (mean±SE) of seeds between seasons (*** stands for significant difference, *t* test, P<0.001).

In Experiment 1 (*i.e.*, tannin-fat experiment), seeds were harvested at similar levels in both seasons (Wald  = 2.685, df = 1, *P* = 0.101), while in Experiment 2 (*i.e.*, tannin-protein experiment), seeds were harvested more quickly in autumn than in summer (Wald  = 355.635, df = 1, *P*<0.001; Fig. 1A). In both experiments, significantly fewer seeds were eaten *in situ*; however, more seeds were removed in autumn than in summer (*P*<0.001, chi-square test; Fig. 1B). In Experiment 2, 15.1% of the seeds were left intact *in situ* after 20 days in summer, while very few seeds were left intact during autumn (1.9%); however, very few seeds were left in either season in Experiment 1 (0.3% and 1.0%). Seeds were dispersed further from the plot in autumn than in summer in both Experiment 1 (7.95 m vs. 4.78 m) (*P*<0.001, t-test) and Experiment 2 (8.36 m vs. 3.38 m) (*P*<0.001, t-test) (Fig. 1C).

### Experiment 1: Effect of Tannin and Fat Contents on Seed Fate

**Figure 2:**
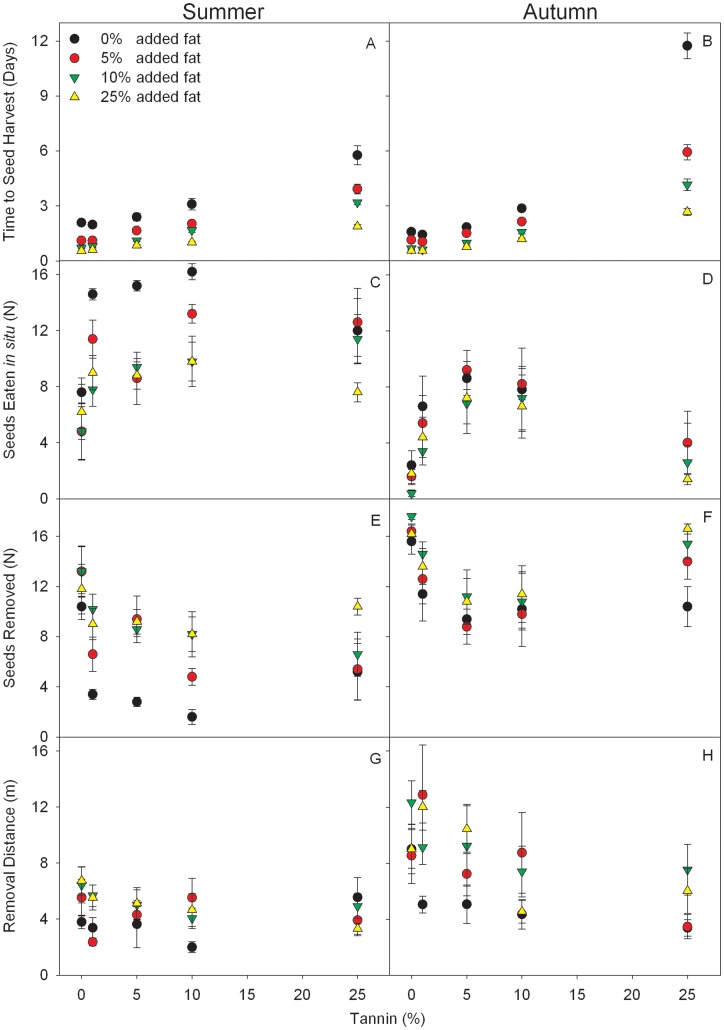
Comparison of fates for seeds with different tannin and fat levels in Experiment 1. The time to seed harvest, seeds removed and eaten *in situ*, and distance to which the seeds were carried by rodents (mean±SE). Seeds removed include seeds cached, eaten after being transported, and missing seeds that were not found within the search area.

After 20 days, 99.7% and 99.0% of the seeds (N = 1800) were harvested by rodents during summer and autumn, respectively. During summer, both tannin and fat affected the time to seed harvest as well as the proportions of seeds that were eaten *in situ* or removed. However, the removal distance of seeds was not affected. Marginally significant interactive effects between tannin and fat were only observed for the time to seed harvest and the proportions of seeds eaten *in situ* (Table 1). Seeds with higher fat and lower tannin concentrations were harvested more quickly by rodents, and were more likely to be removed rather than eaten *in situ*. However, no difference was found for the removal distance of seeds with different tannin and fat concentrations (Fig. 2). In autumn, both tannin and fat content affected the time to seed harvest, the proportions of seeds eaten *in situ* or removed, and removal distance. However, fat content had no effect on the proportion of seeds eaten *in situ* (Table 1). In addition, interactive effects between tannin and fat were only observed for the time to seed harvest (Table 1). Seeds with higher fat content were harvested more quickly, and were more likely to be removed and transported further (Fig. 2). Seeds with higher tannin concentrations were harvested more slowly than seeds with lower tannin concentrations. Furthermore, seeds with both high and low tannin concentrations were more likely to be removed rather than eaten *in situ* than seeds with medium tannin concentrations Seeds with high tannin concentrations and low fat concentrations were not transported far (Fig. 2).

**Table 1:** Summary of the general linear model (GLM) results for the time to seed harvest, seeds removed and eaten *in situ*, and distance to which the seeds were carried by rodents in Experiment 1.

	Summer				Autumn				
	df	MS	F	*P*	df	MS	F	*P*	
**Time to seed harvest**									
Fat	3	356.288	5.858	**0.011**	3	652.513	31.498	**0.000**	
Tannin	4	413.722	11.350	**0.000**	4	1748.348	25.610	**0.000**	
Plot#	4	266.776	3.013	0.043	4	230.404	3.287	0.041	
Fat×Tannin	12	16.927	1.942	0.052	12	218.221	11.548	**0.000**	
Fat×Plot	12	60.822	6.977	0.000	12	20.716	1.096	0.385	
Tannin×Plot	16	36.451	4.181	0.000	16	68.268	3.613	0.000	
Fat×Tannin×Plot	48	8.717	6.887	0.000	48	18.896	7.550	0.000	
**Seeds eaten ** ***in situ*** *									
Fat	3	121.253	18.479	**0.000**	3	21.667	2.383	0.120	
Tannin	4	119.135	6.940	**0.002**	4	153.040	13.496	**0.000**	
Plot	4	45.035	2.699	0.081	4	147.965	9.079	0.000	
Fat×Tannin	12	13.828	1.963	**0.050**	12	2.000	0.484	0.914	
Fat×Plot	12	6.562	0.932	0.524	12	9.092	2.200	0.027	
Tannin×Plot	16	17.166	2.437	0.009	16	11.340	2.744	0.004	
**Seeds removed** *									
Fat	3	131.770	20.552	**0.000**	3	35.707	4.047	**0.033**	
Tannin	4	122.110	7.283	**0.002**	4	138.460	11.668	**0.000**	
Plot	4	45.685	2.842	0.073	4	141.310	8.231	0.001	
Fat×Tannin	12	12.803	1.803	0.075	12	5.540	1.573	0.132	
Fat×Plot	12	6.412	0.903	0.551	12	8.823	2.506	0.012	
Tannin×Plot	16	16.766	2.361	0.011	16	11.866	3.370	0.001	
**Seed removal distance**									
Fat	3	25.982	1.838	0.172	3	303.962	4.185	**0.029**	
Tannin	4	45.980	1.740	0.179	4	319.655	4.028	**0.017**	
Plot	4	27.312	1.782	0.278	4	147.543	1.638	0.240	
Fat×Tannin	12	22.373	0.884	0.567	12	90.708	1.468	0.165	
Fat×Plot	12	12.555	0.496	0.909	12	73.368	1.187	0.315	
Tannin×Plot	16	26.786	1.057	0.414	16	81.147	1.315	0.221	
Fat×Tannin×Plot	44	25.520	1.052	0.388	45	63.260	1.233	0.157	

The degrees of freedom (df), means square (MS), *F*-value (F), and statistical significance level (*P*) for each effect and their interaction are presented.

# : Plot effects were taken as a random factor.

* : Not enough replications to test the 3-factor interactive effects.

### Experiment 2: Effect of Tannin and Protein Contents on Seed Fate

**Figure 3:**
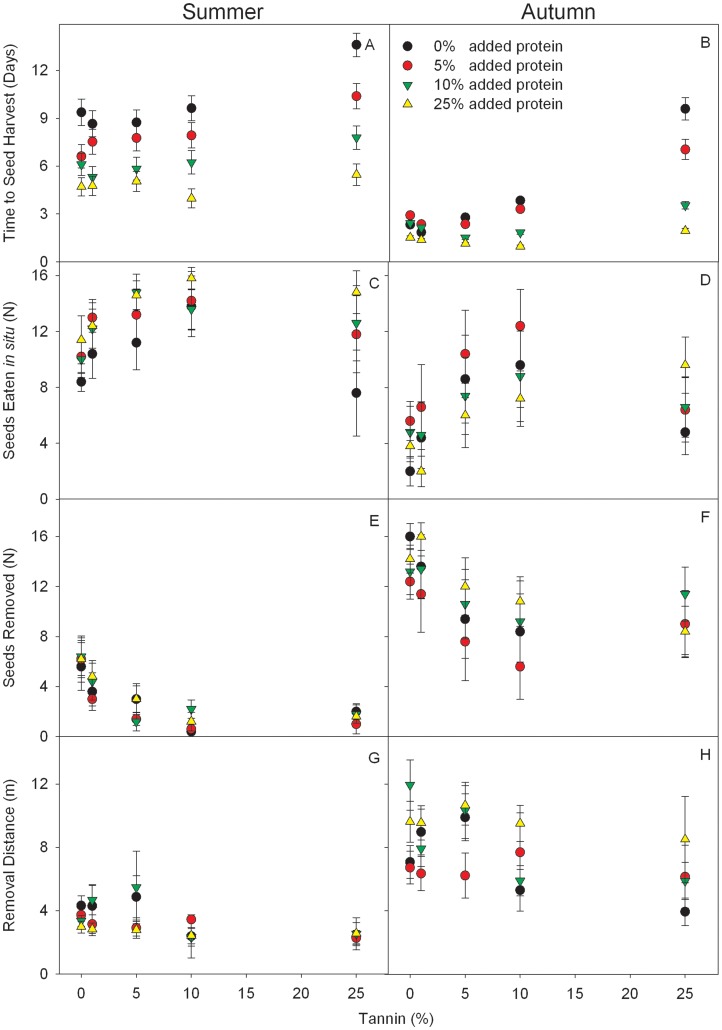
Comparison of fates for seeds with different tannin and protein levels in Experiment 2. The time to seed harvest, seeds removed and eaten *in situ*, and distance to which the seeds were carried by rodents (mean±SE). Seeds removed include seeds cached, eaten after being transported, and missing seeds that were not found within the search area.

After 20 days, 84.9% and 98.1% of the seeds (N = 1800) were harvested by the rodents in summer and autumn, respectively. In summer, tannin had a significant effect on the time to seed harvest and the proportion of seeds eaten *in situ* or removed. However, removal distance was not affected. Protein concentrations only had an effect on the time to seed harvest and the proportion of seeds eaten *in situ* (Table 2). Seeds with higher protein and lower tannin concentrations were harvested by rodents much more quickly. Furthermore, seeds with higher protein concentrations were more likely to be eaten *in situ*, while seeds with low tannin concentrations were more likely to be removed (Fig. 3). No difference was found for the removal distance among seeds with different tannin and protein concentrations (Fig. 3). In autumn, tannin only had an effect on the time to seed harvest, while protein concentrations affected the time to seed harvest and removal distance of seeds (Table 2). Seeds with lower tannin concentrations and higher protein concentrations were harvested more quickly in both seasons (Fig. 3). Seeds with lower tannin concentrations were more likely to be removed and less likely to be eaten *in situ* only in summer only. In comparison, seeds with higher protein concentrations were transported to further distances in autumn only (Fig. 3). Interactive effects between tannin and protein were only found for the time to seed harvest during autumn (Table 2).

**Table 2:** Summary of the general linear model (GLM) results for the time to seed harvest, seeds removed and eaten *in situ*, and distance to which the seeds were carried by rodents in Experiment 2.

	Summer				Autumn			
	df	MS	F	*P*	df	MS	F	*P*
**Time to seed harvest**								
Protein	3	2289.056	13.775	**0.000**	3	680.599	10.318	**0.001**
Tannin	4	474.947	9.219	**0.000**	4	836.784	10.581	**0.000**
Plot#	4	12299.735	68.176	0.000	4	595.893	4.803	0.007
Protein×Tannin	12	62.519	1.677	0.102	12	161.386	7.694	**0.000**
Protein×Plot	12	166.176	4.457	0.000	12	65.965	3.145	0.002
Tannin×Plot	16	51.521	1.382	0.191	16	79.081	3.770	0.000
Protein×Tannin×Plot	48	37.286	2.004	0.000	48	20.976	7.004	0.000
**Seeds eaten ** ***in situ*** *								
Protein	3	53.987	4.427	**0.026**	3	34.493	1.730	0.214
Tannin	4	56.325	3.387	**0.035**	4	110.315	2.303	0.103
Plot	4	143.775	6.392	0.003	4	148.015	2.677	0.067
Protein×Tannin	12	6.012	0.949	0.508	12	14.102	1.123	0.364
Protein×Plot	12	12.195	1.925	0.055	12	19.935	1.588	0.127
Tannin×Plot	16	16.631	2.625	0.005	16	47.909	3.816	0.000
**Seeds removed** *								
Protein	3	4.067	0.298	0.827	3	43.707	2.433	0.115
Tannin	4	84.015	15.013	**0.000**	4	126.465	2.081	0.131
Plot	4	17.340	1.095	0.397	4	121.165	1.849	0.168
Protein×Tannin	12	2.208	0.645	0.793	12	8.998	0.681	0.760
Protein×Plot	12	13.667	3.993	0.000	12	17.965	1.361	0.218
Tannin×Plot	16	5.596	1.635	0.096	16	60.771	4.602	0.000
**Seed removal distance**								
Protein	3	0.845	0.190	0.902	3	381.139	4.938	**0.013**
Tannin	4	3.626	0.466	0.760	4	74.025	1.499	0.232
Plot	4	13.534	1.693	0.205	4	56.142	0.704	0.607
Protein×Tannin	12	2.890	0.780	0.667	12	35.231	0.678	0.765
Protein×Plot	11	4.294	1.195	0.336	12	86.239	1.657	0.103
Tannin×Plot	16	7.805	2.172	0.037	16	50.435	0.964	0.508
Protein×Tannin×Plot	20	3.438	0.686	0.835	38	53.742	1.197	0.204

The degrees of freedom (df), means square (MS), *F*-value (F), and statistical significance level (P) of each effect and their interaction are presented.

# : Plot effects were taken as a random factor.

* : Not enough replications to test the 3-factor interactive effects.

## Discussion

In the current study, tannin, fat, and protein all showed significant effects on rodent foraging behavioral processes. In general, rodents preferred seeds with low tannin and high fat/protein content (Fig. 2, 3). Although only a few interactive effects of fat/protein and tannin were recorded in our study (Table 1, 2), higher concentrations of both fat and protein attenuated the exclusion of seeds with higher tannin concentrations by rodents, especially increasing rodent harvest velocity, *i.e.*, reduction in the time to seed harvest (Fig. 2, 3).

It has been confirmed that dietary tannins can reduce digestion and inhibit assimilation in rodents [11], [20]; hence, it is logical that rodents preferentially select high fat/protein diets to compensate for the negative effects of tannin. Most of the previous studies generally used different species of natural seeds to evaluate the possible effects of fat and/or protein on rodent foraging preferences with respect to seeds with different tannin content levels. Consequently, it was difficult to differentiate the effects of protein and/or fat because varied seed traits were combined [5], [12], [17]. However, 2 other studies [18], [19] used artificial food items to examine the effects of both tannin and fat/protein on foraging preferences in free-ranging scatter-hoarding squirrels. Both studies showed that squirrels preferred foods with low tannin content; these results were supported by the results of our study. Fat is a crucial source of energy that directly affects the survival and reproduction of animals. Most existing studies have shown that rodents prefer seeds/foods with higher fat content [5], [21], [22]. Therefore, the addition of fat may attenuate the exclusion of rodents from high tannin content food [18], as shown in the current study. While protein is extremely important for the growth, reproduction and survival of rodents [23], [24], they usually select food that contains appropriate protein content on the basis of physical requirements, rather than selecting food of the highest available protein content [17], [25]. This is because a critical range of dietary protein levels exists for the normal growth and maintenance of animals [26], [27]. Thus, we found an attenuation effect of protein addition on tannin exclusion by rodents in this study. In comparison, Barthelmess (2001) found that protein had no effect on foraging preference of scatter-hoarding rodents for foods with different tannin content [19].

Our results strongly support that both tannin and fat/protein had a significant effect, as well as some interactive effects, on the seed harvesting process, (Table 1, 2). These results indicate that the decision to harvest a seed is extremely important from both rodent and plant perspectives. When deciding whether to harvest a seed, rodents would assess the seed with respect to both defenses (*i.e*., tannin) and nutrients (*i.e*., fat/protein), in addition to other parameters. Hence, the plant must provide an appropriate mixture of seed traits to escape predation or to stimulate seed dispersal by rodents. However, the effects of tannin and fat/protein on rodent preference vary according to foraging processes (*i.e.*, whether to eat seeds *in situ* or remove away, as well as decisions on how far seeds should be transported). In addition, the effects significantly varied between summer and autumn (Table 1, 2, Fig. 1, 2). One explanation may be that rodents were much more abundant in autumn than in summer (trap success, 12.2% vs. 5.8%). Many studies have demonstrated that rodent abundance has a significant effect on rodent foraging behavior, and consequently the intensity of predation on plant seeds [28]–[30]. Barthelmess (2001) found similar variations in preference by squirrels with respect to different tannin and protein foods between seasons, suggesting food availability as a possible explanation [19].

Some plants are believed to have evolved the ability to manipulate the behavior of scatter-hoarding animals to increase the likelihood that seeds will be stored; hence, tannin and nutrient contents might represent 2 important seed traits in this process [31]. Tannins exist in many plant seeds, and the influence of tannin levels on the selection of seeds by scatter-hoarding rodents has attracted much attention. As a result, many studies have theorized that rodents prefer to feed on low-tannin items over high-tannin ones [13], [18], [19], [32], but see [5], [33], [34]. Fat and protein, which are 2 important seed nutrient indicators, are also believed to have significant effect on rodent foraging behavior [5], [16]–[18]. For many species of seeds dispersed by scatter-hoarding rodents, high tannin content is usually accompanied with high fat or energy content [5], [13]. For instance, many studies have indicated that rodents prefer red oak acorns with high tannin content in place of white oak acorns with low tannin content, with an emphasis on high fat content or big size in red oak acorns [5], [33], [34]. Barthelmess (2001) also discussed that physiological competence, and nutrient complementarity, may explain why squirrels consume tannin in natural diets, despite a preference for low tannin dough balls demonstrated in the researcher’s study results [19]. In our study forest, the preferences of rodents for natural seeds were negatively correlated with tannin content, but positively correlated with protein content; however, fat content did not have an effect on rodent preferences (unpublished data). Although obvious differences were observed in tannin (0–26.48%), fat (4.09–36.53%) and protein (4.56–28.88%) contents in the seeds of 11 common native species (Table 3), no correlations were observed between tannin and fat/protein contents or between tannin versus total fat and protein content (Pearson’s correlation, *P*>0.2). Most seeds of the 11 species were dispersed by wind or frugivorous birds, with only seeds of *Pinus armandii* being primarily dispersed by rodents (unpublished data). Thus, the sample size was too small for a similar analysis to be conducted with respect to the rodent-dispersal syndrome. Furthermore, additional seed traits, other than those evaluated in this study, may also influence rodent foraging behavior. For example, indigestible fibers in seeds are believed to act as important antifeedants for scatter-hoarding rodent [35]. However, further studies are required to explore how plants allocate different seed traits in seeds, as well as what are the dietary variables that most strongly promote the hoarding and caching of seeds by rodents, as opposed to direct consumption *in situ*.

**Table 3:** Tannin, fat and protein contents in seeds from 11 common species in the study area.

Family name	Species	DM (g)	Protein (%)	Fat (%)	Tannin (%)
Cucurbitaceae	*Hemsleya pedunculosum*	6.52	25.66	36.53	0.00
Dipsacaceae	*Dipsacus chinensis*	1.01	17.64	24.66	8.32
Iridaceae	*Iris bulleyana*	1.60	10.98	20.80	26.48
Pinaceae	*Abies forrestii*	2.51	9.00	23.76	25.74
Pinaceae	*Pinus armandii*	24.90	11.88	19.34	1.04
Pinaceae	*Pinus densata*	0.82	28.88	21.30	0.77
Podophyllaceae	*Sinopodophylum hexandrum*	2.83	21.65	16.61	6.40
Ranunculaceae	*Anemone sp.*	0.89	15.53	9.78	1.95
Ranunculaceae	*Thalictrum uncatum*	0.52	13.49	23.58	7.30
Rosaceae	*Cotoneaster sp.*	3.26	4.56	4.09	0.94
Rosaceae	*Rosa omeiensis*	4.89	9.78	9.37	6.54

DM, dry mass of 100 seeds.

The mutualistic interaction between scatter-hoarding rodents and associated seed plants appears to have originated as early as the Paleocene, co-evolving over the last 60 million years [36]. Many plant species with rodent-dispersed seeds appear to have evolved from ancestors with wind-dispersed seeds [36]. To facilitate new seed dispersal mechanisms, seed-producing plants should have evolved a number of traits to accommodate their new partners, such as scatter-hoarding rodents. For example, seeds dispersed by certain rodent and corvids often contain high levels of lipids, proteins, and caloric values, exceeding that present in most wind-dispersed seeds [36]. In the co-evolutionary process between plants and rodents, our results suggest that tannin, fat, and protein content in seeds are all significantly related to scatter-hoarding preferences of rodents during foraging processes. In addition to concentrations of tannin, fat, and protein, numerous other factors in plant seeds may also influence rodent foraging behavior. Therefore, we suggest that rodent foraging preferences should have strong selective pressure on the evolution of plant seeds. Furthermore, we suggest that by clarifying rodent foraging preferences, a better understanding of the evolution of plant seed traits may be obtained because of their strong potential for selective pressure.

## Materials and Methods

### Ethics Statement

This study was carried out in strict accordance with the Guide for the Care and Use of Laboratory Animals of China. The protocol was approved by the Administrative Panel on the Ethics of Animal Experiments of Xishuangbanna Tropical Botanical Garden, Chinese Academy of Sciences (Permit Number: XTBG2009-001). We signed a contract (No. 20090059) with the Shangri-La Alpine Botanical Garden in 2009, and the contract included the permissions to access the study site and conduct this study.

### Study Site

This study was performed during the summer (July and August) and autumn (October and November) of 2009 in a pine forest in the Shangri-La Alpine Botanical Garden, Hengduan Mountains, Yunnan Province, Southwestern China (27° 54′ N, 99° 38′ E, alt. 3456 m). The annual mean temperature is around 5.4°C, and annual rainfall is 625 mm. The forest is mostly natural with limited human disturbance. *Pinus densata* is the dominant tree species, which coexists with several other tree and shrub species. The botanical garden borders the natural forest, thus the rodent community at the study site was not isolated from the natural forest. Rodents in the forest have already been very familiar with our artificial seeds, since we have been conducting the artificial seed experiments for several consecutive years. They are able to discriminate among different tannin/nutrient content artificial seeds with the same shape, even when the differences are minimal (*e.g.,* 0.1% of tannin content level [7]). For more details about the study site, please see [7]. In order to explore the variation of tannin, fat and protein contents of seeds in the forest, we collected 11 common species of seeds for analysis. Seeds of all these 11 species were found to be either eaten *in situ* or removed by rodents in the same forest (unpublished data).The tannin concentration of the 11 species ranged from 0% to 26.48%, with a mean value of 7.77%, while fat and protein content were 19.07% and 15.37%, respectively (Table 3). These data contributed to our design of the amount of tannin, fat and protein content levels in the artificial seeds.

### Rodent Survey

We surveyed rodent abundance across the 2 seasons by using live traps. During the experimental period, live traps were baited with fresh peanuts, to identify the key rodent species that contribute to seed predation and dispersal. To minimize the effect of trapping on the rodent population in the plot where the seeds were released, traps were set about 500 m away from the study site, but in the same forest. Traps were checked twice daily (0700 and 1900), and the captured rodents were recorded and taken to the laboratory. All the captured rodents were subsequently released at the sites where they were originally captured after the survey. In total, 312 live traps were set in summer and 237 live traps were set in autumn.

### Study Materials-artificial Seeds

Clay, laboratory chow (protein: 19.17%, fat: 3.12%, starch: 44.50%, and fiber: 2.41%), tannic acid (C_76_H_52_O_46_, molecular mass 1701. 23, Reijinte Chemistry Ltd., Tianjin, China), protein (soybean protein, ∼92.4% and lactoalbumin, ∼1.8%, Baorui Pharmaceutical Ltd., Guangdong, China), and fat (edible bean oil from local markets) were used to produce the artificial seeds. In this study, clay was used to make the artificial seeds for 2 reasons: (1) it helped with maintaining the other traits as constant while we changed the content level of 1 special trait because the clay contained no tannin, fat, or protein; (2) it allowed us to construct the artificial seeds less fragile and facilitated relocation of the seeds because of its viscosity. Both the clay and laboratory chow were dried (at 120°C and 70°C, respectively) for about 72 h, and ground in a mortar until they passed through a 1 mm screen. The clay powder was mixed with different proportions of laboratory chow, tannic acid, and protein (or fat), after which it was thoroughly homogenized, and water was added until each batch had a doughy consistency. Artificial seeds with a diameter of 15 mm were then made from the batches, and they contained 40% laboratory chow and 60% clay, with variable tannin and fat (or protein) contents. A 15-cm-thin steel thread with a small red plastic tag was connected to each artificial seed to allow the fate of the seed to be tracked more easily. For more details, please refer to [7].

### Experiment 1: Seeds with Different Tannin and Fat Concentrations

We made 20 different treatments of artificial seeds with 5 levels of tannin concentrations (0%, 1%, 5%, 10%, and 25%) × 4 levels of fat concentrations (0%, 5%, 10%, and 25%). Five plots (2 m × 2 m) separated by >50 m were established. At each plot, we located 9 seed release points in a 3 × 3 grid, with about 1 m between points. Each circle-shaped point was about 15 cm in diameter, and the seeds were placed along the circle with the tags located outwards. In summer and autumn, 40 labeled seeds (2 seeds × 20 treatments) were randomly placed at each seed release point, resulting in a total of 360 artificial seeds per plot (40 seeds × 9 seed release points). Each seed treatment was represented by 90 seeds spread evenly across the 5 plots (18 seeds per plot). In total, 1800 seeds (90 seeds × 20 seed treatments) were placed in the plots per season.

After the tagged seeds were placed in the plots, each seed source was checked to record the seeds harvested by rodents. We searched the ground around each seed-placement plot after days 1, 2, 3, 4, 6, 8, 12, 16, and 20. Within 20 m of the plot, we searched the area in every direction to relocate the removed seeds. When we found a seed carried by a rodent, we carefully examined the seed status, and recorded the exact location, including the directional angle and distance to the original seed source.

### Experiment 2: Seeds with Different Tannin and Protein Concentrations

Twenty different artificial seed treatments were performed with 5 levels of tannin concentrations (0%, 1%, 5% 10%, and 25%) × 4 levels of protein concentrations (0%, 5%, 10%, and 25%). We conducted the experiment in summer and autumn at the same 5 plots, about 10 days after Experiment 1. All the methods used in this experiment were same as those used in Experiment 1.

### Data Analyses

Seed fates were first divided into 2 categories: (1) harvested by rodents and (2) left intact *in situ*. Harvested seeds included those eaten *in situ* and removed by rodents (*i.e.,* seeds cached, eaten after being transported, and missing seeds that were not found with in the search area), according to the methodology followed in [7]. Only a small number of removed seeds were found cached by rodents in both experiments (Table S1, S2). Thus, the sample size within each treatment of artificial seed was too small to analyze the cached seeds, similar to the removed or eaten seeds. A significant positive relationship was found between the number of seeds that were cached and removed by rodents (Pearson’s correlation: r = 0.654, *P*<0.001); hence, the number of seeds that were removed could be considered as an indicator of seeds cached, to a certain extent.

SPSS 13.0 for Windows was used for statistical analysis. We used the counting process notation to record the number of seeds subjected to each seed fate (*i.e.*, left intact *in situ*, eaten *in situ*, and removed by rodents), and the number of rodents captured in live traps. Thus, Chi-square tests were used to test the difference in trap success and composition of rodent species between seasons, and the frequency of differences among seed fates between the 2 seasons. Cox regression model was used to test the differences in the time to seed harvest between seasons. A general linear model (GLM) was used to analyze the time to seed harvest, number of seeds eaten *in situ* and removed, and the removal distance of seeds, with tannin and fat (or protein) as the 2 fixed factors and plot effect as a random factor.

## Supporting Information

Table S1 **Summary results of number of seeds cached and removed by rodents in Experiment 1.**
(DOC)

Table S2 **Summary results of number of seeds cached and removed by rodents in Experiment 2.**
(DOC)
