# Supplementary material for: Effects of Fat and Protein Levels on Foraging Preferences of Tannin in Scatter-Hoarding Rodents
Source: PLoS One. 2012 Jul 10;7(7):e40640. doi: 10.1371/journal.pone.0040640 (PMC3393693; doi:10.1371/journal.pone.0040640)
Supplement: Table S1 — Summary results of number of seeds cached and removed by rodents in Experiment 1. (DOC) [file pone.0040640.s001.doc]

**Table S1 Summary results of number of seeds cached and removed by rodents in Experiment 1 (Five plots data combined).**

|  |  |  | Summer | | Autumn | |
| --- | --- | --- | --- | --- | --- | --- |
| No. of Treatment | Fat Added | Tannin Added | Cached | Removed | Cached | Removed |
| 1 | 0% | 0% | 1 | 52 | 13 | 78 |
| 2 | 0% | 1% | 0 | 17 | 17 | 57 |
| 3 | 0% | 5% | 0 | 14 | 15 | 47 |
| 4 | 0% | 10% | 0 | 8 | 12 | 51 |
| 5 | 0% | 25% | 5 | 26 | 16 | 52 |
| 6 | 5% | 0% | 0 | 66 | 11 | 82 |
| 7 | 5% | 1% | 0 | 33 | 11 | 63 |
| 8 | 5% | 5% | 0 | 47 | 14 | 44 |
| 9 | 5% | 10% | 0 | 24 | 7 | 49 |
| 10 | 5% | 25% | 1 | 27 | 13 | 70 |
| 11 | 10% | 0% | 1 | 66 | 14 | 88 |
| 12 | 10% | 1% | 0 | 51 | 12 | 73 |
| 13 | 10% | 5% | 0 | 43 | 9 | 56 |
| 14 | 10% | 10% | 1 | 41 | 3 | 54 |
| 15 | 10% | 25% | 1 | 33 | 9 | 77 |
| 16 | 25% | 0% | 0 | 59 | 6 | 81 |
| 17 | 25% | 1% | 0 | 45 | 6 | 68 |
| 18 | 25% | 5% | 0 | 46 | 12 | 54 |
| 19 | 25% | 10% | 0 | 41 | 4 | 57 |
| 20 | 25% | 25% | 0 | 52 | 9 | 83 |
| total |  |  | 10 | 791 | 213 | 1284 |
