# Supplementary material for: Effects of Fat and Protein Levels on Foraging Preferences of Tannin in Scatter-Hoarding Rodents
Source: PLoS One. 2012 Jul 10;7(7):e40640. doi: 10.1371/journal.pone.0040640 (PMC3393693; doi:10.1371/journal.pone.0040640)
Supplement: Table S2 — Summary results of number of seeds cached and removed by rodents in Experiment 2. (DOC) [file pone.0040640.s002.doc]

**Table S2 Summary results of number of seeds cached and removed by rodents in Experiment 2 (Five plots data combined).**

|  |  |  | Summer | | Autumn | |
| --- | --- | --- | --- | --- | --- | --- |
| No. of Treatment | Protein Added | Tannin Added | Cached | Removed | Cached | Removed |
| 1 | 0% | 0% | 9 | 28 | 17 | 80 |
| 2 | 0% | 1% | 4 | 18 | 25 | 68 |
| 3 | 0% | 5% | 6 | 15 | 7 | 47 |
| 4 | 0% | 10% | 1 | 2 | 8 | 42 |
| 5 | 0% | 25% | 5 | 10 | 3 | 45 |
| 6 | 5% | 0% | 10 | 31 | 4 | 62 |
| 7 | 5% | 1% | 4 | 15 | 16 | 57 |
| 8 | 5% | 5% | 3 | 7 | 15 | 38 |
| 9 | 5% | 10% | 0 | 3 | 5 | 28 |
| 10 | 5% | 25% | 3 | 5 | 9 | 45 |
| 11 | 10% | 0% | 10 | 32 | 16 | 66 |
| 12 | 10% | 1% | 9 | 22 | 21 | 67 |
| 13 | 10% | 5% | 1 | 6 | 10 | 53 |
| 14 | 10% | 10% | 1 | 11 | 17 | 46 |
| 15 | 10% | 25% | 3 | 9 | 10 | 57 |
| 16 | 25% | 0% | 8 | 31 | 21 | 71 |
| 17 | 25% | 1% | 7 | 24 | 25 | 80 |
| 18 | 25% | 5% | 4 | 15 | 16 | 60 |
| 19 | 25% | 10% | 3 | 6 | 18 | 54 |
| 20 | 25% | 25% | 3 | 8 | 12 | 42 |
| total |  |  | 94 | 298 | 275 | 1108 |
